# Effect of the epiphytic bacterium *Bacillus* sp. WPySW2 on the metabolism of *Pyropia haitanensis*

**DOI:** 10.1007/s10811-017-1279-z

**Published:** 2017-12-02

**Authors:** Yuqin Xiong, Rui Yang, Xiaoxiao Sun, Huatian Yang, Haimin Chen

**Affiliations:** 1 Key Laboratory of Marine Biotechnology of Zhejiang Province, Ningbo University, No. 818 Fenghua Road, Post Box 71, Ningbo, Zhejiang 315211 China; 2 Li Dak Sum Yip Yio Chin Kenneth Li Marine Biopharmaceutical Research Center, Ningbo University, Ningbo, Zhejiang 315211 China

**Keywords:** *Bacillus* sp., *Pyropia haitanensis*, Phycosphere, Co-culture, Metabolomics

## Abstract

A variety of different symbiotic microbial communities are harbored on the surface of seaweeds, the interactions of which depend upon nutritional exchanges between the microbes and the hosts. Metabolomic profiling is able to provide a comprehensive and unbiased snapshot of the metabolites associated with seaweed–microbe interactions. In this study, the relationships between phycosphere bacteria and the red alga *Pyropia haitanensis* were investigated on a metabolomic basis using gas chromatography–mass spectrometry, and the pathways of the interactions between the seaweed and its associated phycospheric microbes were revealed. *Bacillus* sp. WPySW2, one bacterial species isolated from the phycosphere of *Pyropia* species, had a significant influence on the metabolomic profile of the algae. Some of the intracellular metabolites such as phenylalanine, leucine, isoleucine, valine, proline, tyrosine, threonine, octadecanoic acid, hexadecanoic acid, and citric acid were downregulated in the thalli of *P*. *haitanensis* when it was co-cultured with *Bacillus* sp. WPySW2, while several special metabolites including melibiose, serine, glycerol-3-phosphate, galactosylglycerol, and alanine were upregulated. The results demonstrated that *P*. *haitanensis* grew better when it was co-cultured with *Bacillus* sp. WPySW2 at 20 °C. In conclusion, several main intracellular metabolites were downregulated and upregulated, which might have facilitated bacterial colonization.

**Electronic supplementary material:**

The online version of this article (10.1007/s10811-017-1279-z) contains supplementary material, which is available to authorized users.

## Introduction

In the marine environment, the surfaces of seaweeds harbor a variety of different symbiotic microbial communities that influence the development and physiological status of their host (Wahl et al. 2012). The seaweeds provide photosynthate to their microbial neighbors in return for a habitat that protects the host from desiccation or light, gases, and/or nutrient stress and modulates the host’s interaction with additional foulers, consumers, or pathogens (Hawksworth 1988; Wahl et al. 2012). Other symbiotic interactions exist. For example, the biomass of microalga *Dunaliella* sp. accumulated with the presence of two bacteria strains affiliated to *Alteromonas* sp. and *Muricauda* sp*.*, and the bacteria helped the algae accumulate nitrogen (Chevanton et al. 2013). These symbiotic relationships suggest that seaweeds and their epiphytic bacteria interact in a type of substance metabolism, complementing the living environment to form a specific environment (Egan et al. 2013), defined as a “phycosphere” (Bell and Mitchell 1972). Some symbiotic bacteria isolated from seaweeds could produce secondary materials that exhibited antibacterial activity (Vijayalakshmi 2015; Albakosh et al. 2016).

In the rhizosphere, the gram-positive bacterium *Bacillus* species are important symbionts of the terrestrial plant roots (Vlamakis et al. 2013). *Bacillus* species also commonly inhabit the marine environments (Olmos-Soto 2014), and have been found to be dominant in the phycosphere of *Pyropia a*nd *Ulva* species (Yang et al. 2008; Shen et al. 2013). The effects of troponoid compounds derived from the genus *Phaeobacter* on an algal–bacterial symbiosis were also reported (Seyedsayamdost et al. 2011). Seaweeds themselves also secrete surface-associated secondary metabolites, enabling chemical defense against bacterial colonization. It was reported that macroalgae regulate the behavior of algal surface epiphytic bacteria directly through bromine and chlorine, 1,1,3,3-tetrabromo-2-heptanone, and furanones, sesquiterpenes (Vairappan et al. 2008; Nylund et al. 2010; Persson et al. 2011; Fernandes et al. 2012; Carvalho et al. 2017), oxidase (Weinberger et al. 2011), proline (Lachnit et al. 2010; Saha et al. 2014), free fatty acid derivatives, (Lion et al. 2006) and bromophycolides (Lane et al. 2009). For microbial–microbial interactions, probiotics or symbiotic bacteria act as the trigger of chemical defenses of the other microbial communities and alter the microbial flora in the phycosphere (Wahl et al. 2012). Enzymes such as *N*-acyl homoserine lactone (AHL)-acylase and AHL-lactonase are produced by *Bacillus* spp., which might act as pathogen community inhibitors (Cao et al. 2012).

*Pyropia haitanensis* occurs locally in the Zhejiang and Fujian provinces in China which is primarily cultivated in these areas and is of great commercial importance (Chen et al. 2011). Many pathogenic microbes that cause serious disease in *Pyropia* species have been reported, including *Cobetia marina*, *Pseudoalteromonas citrea*, *Pythium porphyrae*, and so on (Takahashi et al. 1977; Yan et al. 2002, 2008; Huang and Yan 2010). However, the pathogenic bacteria might not be the only cause of algal disease. Shen et al. (2013) reported that many bacterial strains isolated from the healthy thalli of *P*. *haitanensis* were highly similar to these virulent algal pathogens. The species and biomass of bacteria in the *Pyropia* phycosphere were found to vary with the algal species and culture conditions, the physiological status (health and disease), and algal development stages (Yang et al. 2008). The common bacteria species found in phycosphere include *Pseudoalteromonas*, *Psychrobacteria*, and *Bacillus* (Yan et al. 2002; Wang et al. 2011). Fang et al. (2009) reported that 3 PKS I positive *Bacillus* strains (WPhG3, WPySW1, and WPySW2) isolated from the phycosphere of cultivated *P*. *haitanensi*s had powerful antibacterial activities, similar to many *Bacillus subtilis* strains reported in some terrestrial plants (Chung et al. 2015). *Bacillus* species were also able to increase the content of chlorophyll in rice, increase the fresh weight and dry weight of the plant, enhance the activities of protection enzymes, advance the resistance to adversity by improving adjustability, and promote the growth of seedlings (Cai et al. 2005; Yang et al. 2010). Song et al. (2014) reported that the inhibition of calcification reaction of *Pleurochrysis carterae* by the phycospheric *Psedudoalteromonas* sp. NPyS3 was significantly greater than that of lower pH.

Metabolomic profiling provides an applicable and unbiased snapshot of metabolites belonging to predefined groups, including organic acids, fatty acids, sugars, and amino acids (Agarrwal et al. 2014). Analytical techniques such as gas chromatography–mass spectrometry (GC-MS) have been successfully applied to investigate and profile the metabolic variation associated with different conditions as for example, the responses of cassava (*Manihot esculenta*) roots during postharvest physiological deterioration (Uarrota et al. 2014). Therefore, metabolomic analysis has been shown to be a powerful tool for gaining new insights and a better understanding of the metabolic responses to biotic factors. In this study, we investigated the relationship between phycosphere bacteria and *P. haitanensis* by detecting the metabolite basis with GC-MS techniques and revealing the pathways of the interactions between the seaweed and associated phycosphere microbes.

## Materials and methods

*Bacillus* sp. WPySW2 was isolated from the phycosphere of rotten *Pyropia* species cultivated in Wenzhou area and preserved in the Key Laboratory of Marine Biotechnology of Zhejiang Province, Ningbo University, China. The NCBI code number of 16S sRNA sequence is EU863221. The bacteria were incubated in 250-mL flasks containing 100 mL 2216E medium (Patrick 1978) and cultured at 37 °C and 140 rpm for 12 h. The cells were diluted to the concentration of 10^8^ cells mL^−1^, centrifuged for 1 min at 5000×*g* to remove the 2216E medium, and suspended in a small amount of sterile cultured medium (*v*/*v*: mixed solution KNO_3_ 100 g L^−1^ and KH_2_PO_4_ 10 g L^−1^: sterile seawater = 1:1000; Zhou et al. 2012).

*Pyropia haitanensis* thalli were collected in October 2014 from the culture area of the Dongtou coast, Zhejiang Province, China. After being naturally dried, the thalli were sealed and stored at − 20 °C. The samples were recovered with seawater at 20 °C for 24 h and examined under a microscope. Healthy thalli were cleaned three times with a soft brush in sterile seawater and soaked in 0.7% KI (*w*/*v*) for 10 min. The samples were then cleaned three times again using sterile seawater for further use. The cleaned thalli were immersed in a combination of the antibiotics—ampicillin (final concentration 300 μg mL^−1^), kanamycin (final concentration 100 μg mL^−1^), and gentamicin (final concentration 100 μg mL^−1^)—for 18 h and cleaned with sterile seawater (Zhou et al. 2012).

### Determination of *P*. *haitanensis* biomass

The cleaned antibiotic treated algae pieces (ca. 0.2 g) were treated with suspended bacteria (3 × 10^8^ cells mL^−1^) and incubated in 300-mL sterile flasks containing sterile cultured medium. The flasks were left standing at 20 °C for 9 days (light intensity 50–70 μmol photons m^−2^ s^−1^, L: D = 12 h: 12 h). We measured the fresh weight of the algae every 3 days and calculated the relative growth rate of the algae using the formula: relative growth rate (RGR) (%/day) = [Ln (M_t_/M_o_)/t] × 100 (Kain 1987).

### *P*. *haitanensis*–*Bacillus sp.* WPySW2 co-cultures

The cleaned thalli (0.2 g) were selected randomly and co-cultured with suspended bacteria (3 × 10^8^ cells mL^−1^) and incubated in 300 mL sterile nutrient enriched seawater. The co-cultured systems (B-Ph) were incubated at 20 °C under a 12 h/12 h light/dark regime with 50–70 μmol photons m^−2^ s^−1^ illumination. The control groups (pure culture of *P. haitanensis*, Ph-C group) were inoculated in the flasks under identical sterile cultured medium and concentration conditions as above.

The experiments were repeated three times and the samples were collected for further analysis. The progress of co-culture was recorded by microscopy. At the end of culture, the thalli were collected and immersed in 0.1% (*w*/*v*) ampicillin for 10 min and thereafter cleaned three to five times with sterile seawater to remove the epiphytic bacteria. The surface water was absorbed, and the samples were frozen with liquid nitrogen and transported to the − 80 °C freezer as quickly as possible.

### Metabolite extraction and metabolite profiling analysis

The metabolites of the thalli (cultured for 3 days) were extracted according to Lisec et al. (2006), with some modifications. Eight repeat samples (100 mg fresh weight per sample) of both the control and the co-culture systems were collected randomly and stored at − 80 °C for metabolite analysis. The samples were ground in liquid nitrogen and transferred into 2-mL centrifuge tubes. In total, 1200 μL of 100% methanol (pre-cooled at − 20 °C) was added and vortexed for 10 s; then 60 μL of ribitol (0.2 mg mL^−1^ stock in dH_2_O) was added as an internal quantitative standard and vortexed for 10 s. The tubes were placed into an ultrasonic processor at 70 °C for 30 min and then centrifuged for 10 min at 11000×*g*. One milliliter supernatant was transferred into 10-mL glass centrifuge tubes. After adding 750 μL chloroform (pre-cooled at − 20 °C) and 1500 μL deionized water (dH_2_O; 4 °C), the tubes were vortexed for 30 s, then centrifuged for 15 min at 3000×*g*. Then, 1000 μL supernatant was transferred into a new Eppendorf tube. The samples were blow-dried with a moderate nitrogen stream. Next, 50 L of 15 mg mL^−1^ methoxyamine pyridine solution was then added, and the mixture was vortexed for 30 s and reacted for 90 min at room temperature. Finally, 50 μL BSTFA reagent (containing 1% TMCS) was added to the mixture and reacted for 60 min at 70 °C. Following the above reactions, the samples were determined for metabolite contents using an Agilent 7890A GC system coupled to an Agilent 5975C inert XL EI/CI mass spectrometric detector (MSD) system (Agilent Technologies, USA). Furthermore, mixed n-alkane standard solutions C_8_–C_20_ and C_21_–C_40_ (Sigma Aldrich) were used for the determination of retention indices (RI).

### GC-MS analysis

Gas chromatography was performed on an HP-5MS capillary column (5% phenyl-95% methylpolysiloxane, 30 m × 250 μm i.d., 0.25-μm film thickness; Agilent J & W Scientific, USA) to separate the derivatives at a constant flow of 1 mL min^−1^ helium. Then, 1 μL of sample was injected in split mode in a 20:1 split ratio by the auto-sampler. The injection temperature was 280 °C, the interface was set to 150 °C, and the ion source was adjusted to 230 °C. The temperature-rise program was initial temperature of 80 °C held for 5 min, thereafter increased at a rate of 20 °C min^−1^ up to 300 °C, and maintained at 300 °C for 6 min. Mass spectrometry was determined using the full-scan method in the range of 35 to 500 m/z.

For the quality control (QC) samples (Sangster et al. 2006), an aliquot (about 40 uL) of all prepared sample extracts was mixed. These QC samples were used to monitor deviations of the analytical results from these pooled mixtures and compare them to the errors caused by the analytical instrument itself. The QC pool was subsequently divided over 10 vials and analyzed regularly throughout the entire analysis batch. Additionally, empty vials (blank injections) were included into the measurement sequence to test for laboratory contamination.

### Data processing

Raw GC-MS data were converted into CDF format (NetCDF) files by Agilent GC/MS 5975 Data Analysis software and subsequently processed using XCMS (www.bioconductor.org), implementing the default settings with the following modifications: xcmsSet (fwhm = 3, snthresh = 3, mzdiff = 0.5, step = 0.1, steps = 2, max = 300) and group (bw = 2, minfrac = 0.3, max = 300). The signal integration area of each metabolite was normalized to the internal standard (ribitol) for each sample. Identification of metabolites using the Automatic Mass Spectral Deconvolution and Identification System (AMIDS) was searched against commercial available databases such as the National Institute of Standards and Technology (NIST) and Wiley libraries. Metabolites were confirmed by comparison of mass spectra and retention indices to the spectra library using a cut-off value of 70% (Dawud et al. 2012).

### Multivariate statistical analysis

For multivariate statistical analysis, the XCMS output was further processed using Microsoft Excel (Microsoft, USA). Finally, the normalized data were imported into Simca-P software (version 11.0, http://www.umetrics . com/simca) for multivariate statistical analyses, including principal component analysis (PCA) and partial least squares-discriminant analysis (PLS-DA). All data were mean-centered and unit variance (UV)-scaled before PCA and PLS-DA were applied in order to guard against overfitting. In this study, a default sevenfold (Leave-1/7th samples-Out) cross validation procedure, as well as 100 random permutation testing, was performed to guard against overfitting of supervised PLS-DA models. *R* ^2^ *X* and *R* ^2^ *Y* represent the fraction of the variance of the *x* and *y* variables explained by the model, while *Q* ^2^ *Y* suggests the predictive performance of the model. The cumulative values of *R* ^2^ *X*, *R* ^2^ *Y*, and *Q* ^2^ *Y* vary from 0 to 1.

These discriminating metabolites were obtained by using a statistically significant threshold of variable influence on projection (VIP > 1.0) values obtained from the PLS-DA model and were further validated by Student’s *t* test. The metabolites with VIP values greater than 1.0 and *p* values less than 0.05 (threshold) were selected as discriminating metabolites between two classes of samples.

### Statistical analysis

Hierarchical cluster analysis (HCA) was performed and visualized using the heatmap package in R (www.r-project.org). Pearson’s product-moment correlation (Pearson’s *r*) was performed to calculate the correlation. Corresponding *p* values and false discovery rate (FDR) (Benjamini and Yekutieli 2001) of each correlation were also calculated using the Cor. test function in R. Identified metabolites were mapped onto general biochemical pathways according to annotation in KEGG (Kyoto Encyclopedia of Genes and Genomes). Metabolic network maps were constructed by incorporating the identified and annotated metabolites using Cytoscape 3.2.0 software (http://www.cytoscape.org/).

## Results

### WPySW2 colonization on the surface of the seaweed

**Fig. 1:**
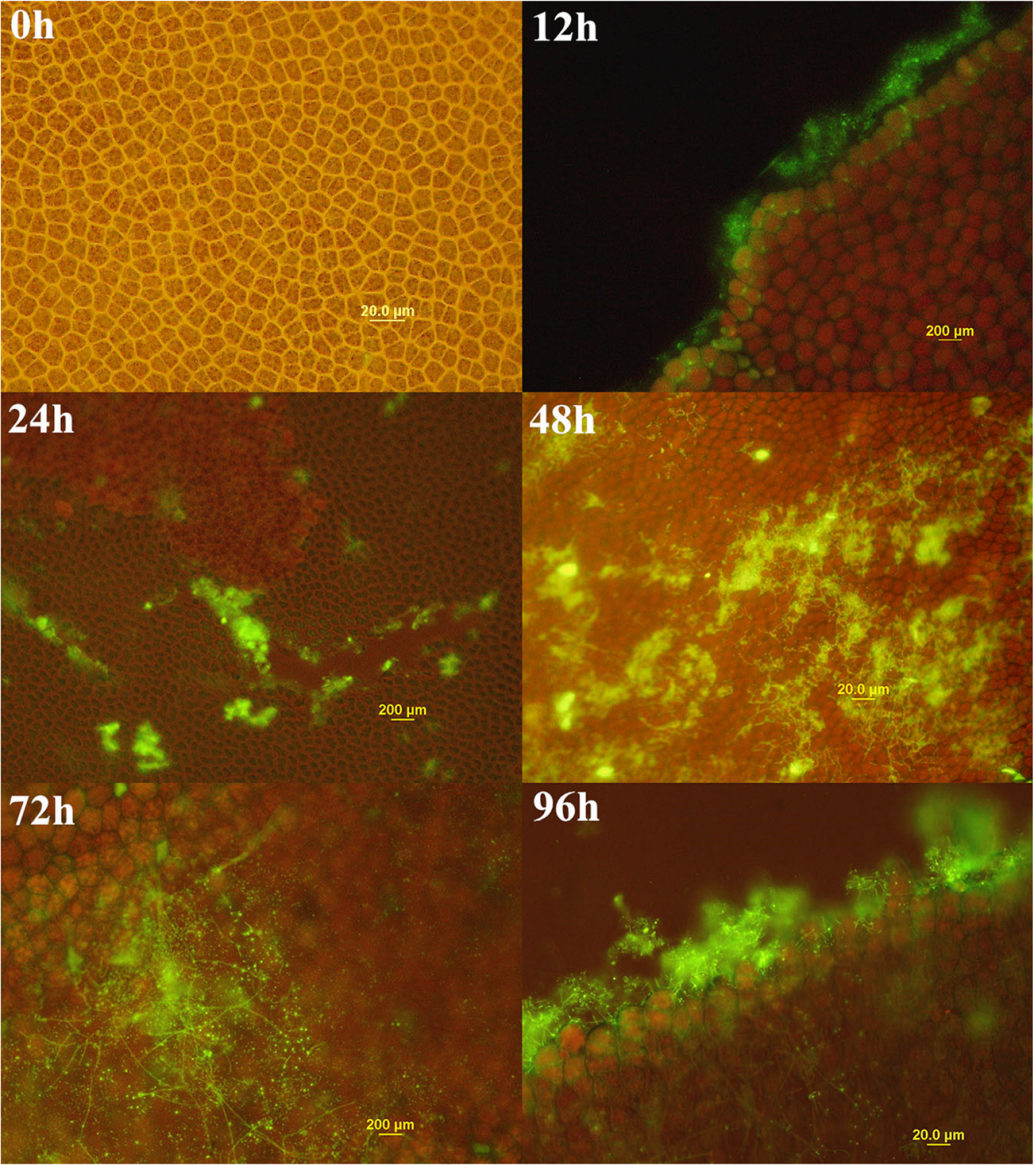
Bacterial colonization on the surface of *P*. *haitanensis* thalli in the co-culture systems

WPySW2 colonized on the surface of the algal thalli when it was co-cultured with *P*. *haitanensis* (Fig. 1), while the surface of the control group remained clean for a long time. After being cultured for 12 h, the bacteria began to contact to the surface of thalli. The small pieces of green fluorescence were blocked, and the bacteria assembled continually as time went by. It was observed that bacteria preferred to accumulate at the edge of the leaves of the wound, which might provide more nutrient for the bacterial growth.

### Relative growth rate of *P*. *haitanensis*

**Fig. 2:**
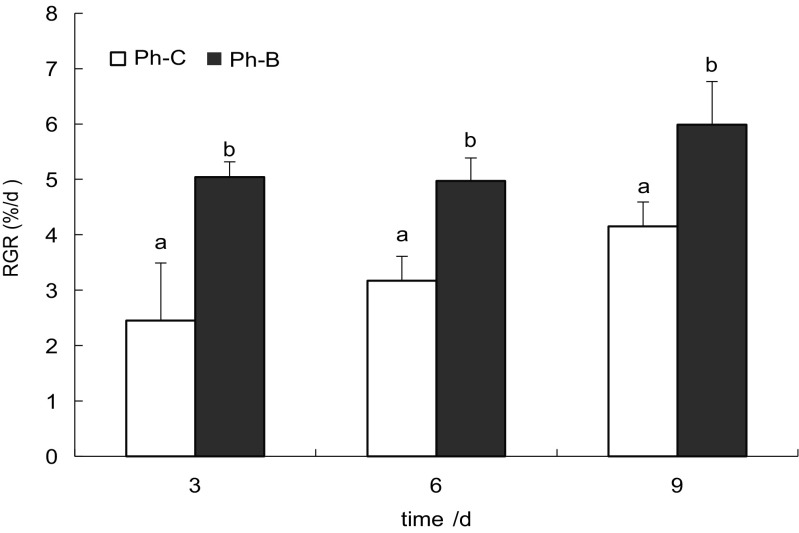
The influence of bacteria on the growth of *Pyropia haitanensis*. Relative growth rates in % day^−1^ of *P*. *haitanensis* algae after 9 days of incubation in the sterile seawater under different conditions (see the “Materials and methods” section). Mean values ± SD (*n* = 3) and the figure indicate significantly increased of the growth rates (two-way ANOVA) driven by bacteria and culture time (*p* < 0.05) (B-Ph: *P. haitanensis* thalli co-cultured with *Bacillus* sp. WPySW2, Ph-C: *P. haitanensis* cultured without bacteria)

The two-way ANOVA results revealed that the relative growth rate (RGR) of *P*. *haitanensis* thalli at 20 °C was significantly promoted by *Bacillus* sp. WPySW2 (*p* < 0.01) and the culture time (*p* < 0.05; Fig. 2), and the bacteria had greater effects. These Gram-positive bacteria exhibited similar features as plant growth hormones. An increasing growth trend was observed in the *P*. *haitanensis* thalli from day 3 to day 9. The highest RGR was obtained in the co-cultured system (B-Ph) at day 9, which was 5.99% day^−1^, while the RGR of the control (Ph-C) group was only 4.15% day^−1^. The RGR at day 3 in the B-Ph group was 2.06 times that in the Ph-C group, while the ratio of RGR between the B-Ph and Ph-C groups declined to 1.57 and 1.44 times at day 6 and day 9.

### Cluster analysis of metabolite profiles

**Fig. 3:**
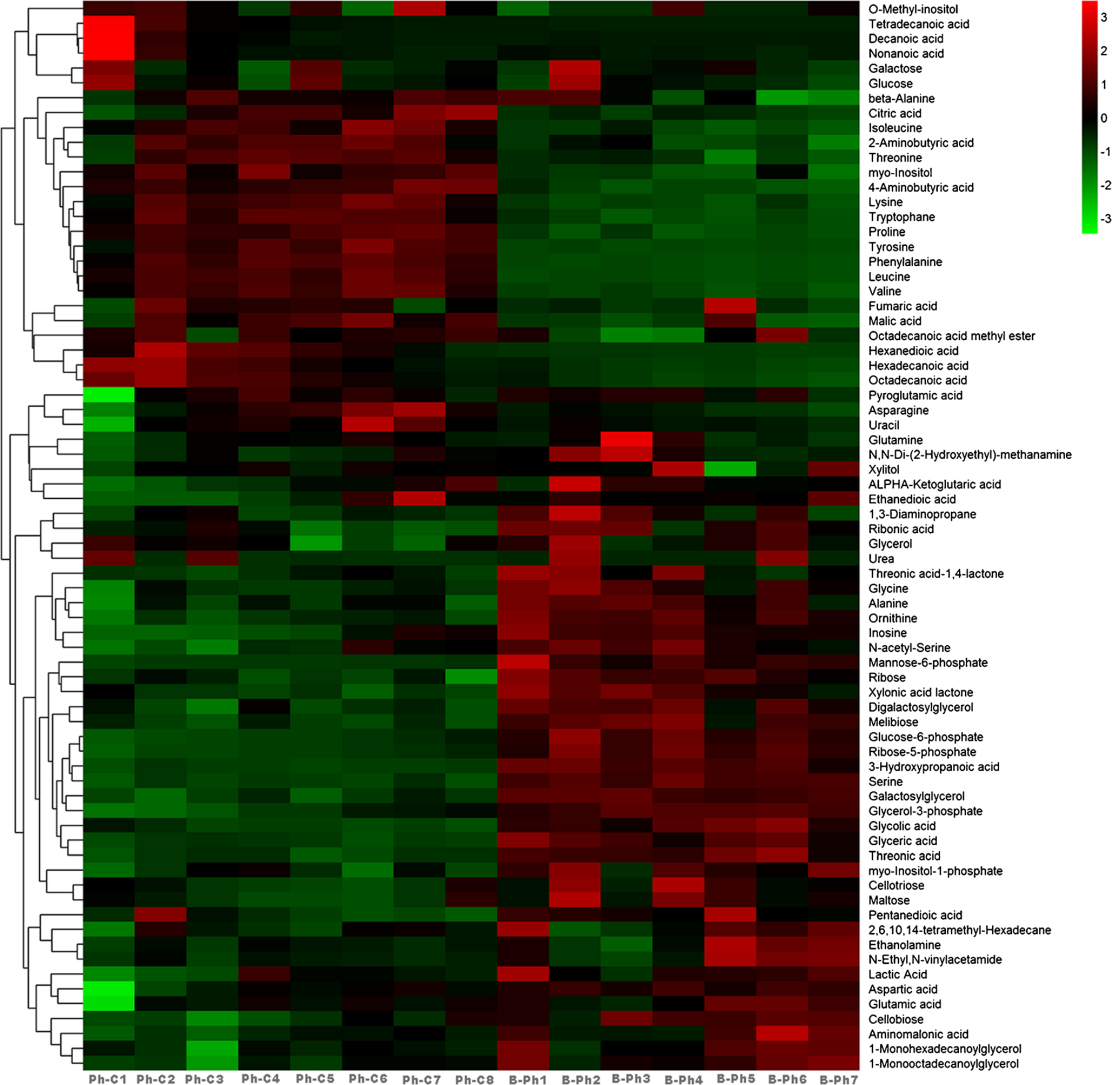
Heatmap of cellular metabolites profile from *P*. *haitanensis* in control (Ph-C) and co-culture samples (B-Ph)

Cluster analysis is a multivariate statistical analysis method to classify samples or indicators. Cluster analysis originated in taxonomy (Zhang et al. 2008; Real et al. 2012; Farag et al. 2012;), and a large number of samples have been classified reasonably using their respective features. Hierarchical clustering analysis (HCA) was performed to classify the control (Ph-C) and co-culture (B-Ph) metabolites. These 72 metabolites covered different primary metabolism pathways and some of the secondary metabolism pathways and were classified into several major metabolite groups (Fig. 3). As shown in Table 1, 15 metabolites varied significantly between the Ph-C and the B-Ph samples. Five metabolites (including serine, melibiose, alanine, galactosylglycerol, and glycerol-3-phosphate) exhibited higher levels in co-culture B-Ph samples, while 10 (including octadecanoic acid, hexadecanoic acid, leucine, valine, citric acid, proline, tyrosine, threonine, phenylalanine, and isoleucine) exhibited lower levels in B-Ph groups.

**Table 1:** Metabolic response of *P*. *haitanensis*

Name	Class	RI^a^	RT^b^	*m*/*z* ^c^	*p* value (B-Ph/Ph-C)^d^	Log (B-Ph/Ph-C)^e^	VIP^f^
Octadecanoic acid	Fatty acid	2247.2	14.8751	341.3	0.000421	− 0.4594	3.4544
Hexadecanoic acid	Fatty acid	2049.7	13.9765	313.3	0.0013	− 0.4845	3.2618
Leucine	Amino acid	1282.9	9.2537	158.1	3.52E−09	− 0.7693	2.8377
Valine	Amino acid	1227.0	8.7082	144.1	7.26E−08	− 0.6022	2.1835
Serine	Amino acid	1373.8	10.0187	218.1	1.97E−10	0.8548	2.1463
Melibiose	Sugar	3204.7	19.9937	204.1	1.31E−05	0.4430	1.8439
Citric acid	Organic acid	1840.9	12.9335	273.1	0.011481	− 0.5293	1.8325
Proline	Amino acid	1307.8	9.4836	142.1	7.76E−09	− 0.6711	1.8059
Glycerol-3-phosphate	Phosphate	1782.6	12.6233	357.1	1.83E−06	1.0526	1.5670
Tyrosine	Amino acid	1960.4	13.5433	218.1	1.47E−06	− 0.6932	1.2171
Threonine	Amino acid	1400.3	10.2324	218.1	0.000274	− 0.1683	1.2001
Phenylalanine	Amino acid	1646.0	11.8477	192.1	8.19E−09	− 0.7601	1.1961
Isoleucine	Amino acid	1304.0	9.4527	158.1	3.47E−05	− 0.3881	1.1672
Galactosylglycerol	Glycols	2352.7	15.3243	217.1	4.47E−08	0.3757	1.1006
Alanine	Amino acid	1112.4	7.3388	190.1	0.00078	0.2380	1.0985

^a^Retention index

^b^Retention time

^c^Quantification mass

^d^Student’s *t* test

^e^Logarithmic transformed fold change

^f^VIP values obtained from PLS-DA

### Metabolite–metabolite correlation analysis

**Fig. 4:**
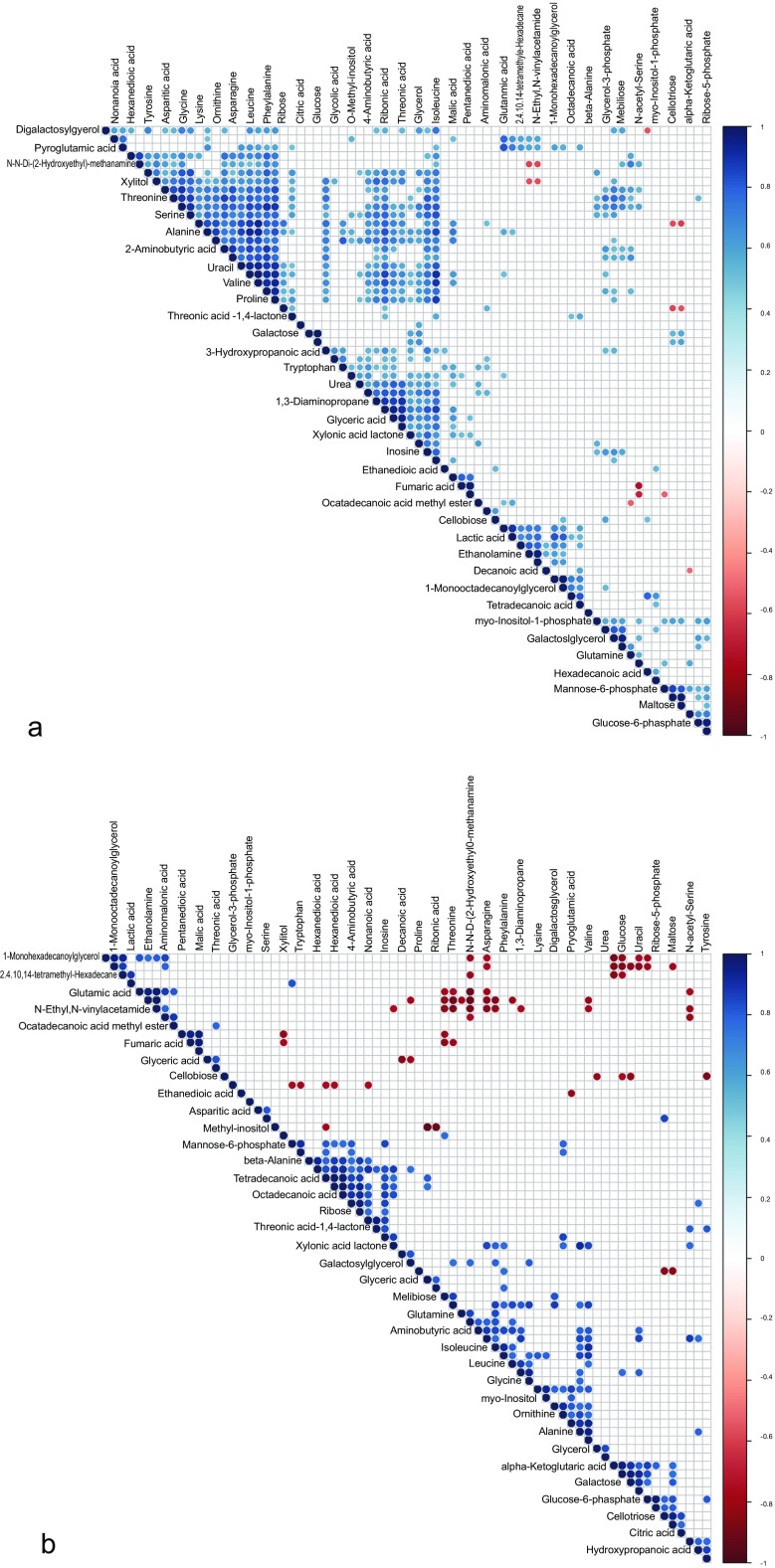
Metabolite–metabolite correlations of samples. **a** Control samples (Ph-C). **b** Co-culture samples (B-Ph)

Pearson’s pairwise correlation coefficients were calculated for each pair of identified metabolites and visualized on a heatmap. Metabolite-metabolite correlations between the tissue of the Ph-C and B-Ph samples showed unique profiles. In the control, a total of 2556 correlations were analyzed, and out of these metabolite correlations, 506 resulted in significant correlation coefficients (*p* < 0.05). Out of these significant correlations, 330 were positive and 176 were negative (Fig. 4a). In the co-culture samples, 2556 correlations were also analyzed, and out of these metabolite correlations, 255 resulted in significant correlation coefficients (*p* < 0.05). Out of these 255 significant correlations, 192 were positive and 63 were negative (Fig. 4b).

### Correlation-based network analysis to identify *P*. *haitanensis*-induced metabolic perturbation

**Fig. 5:**
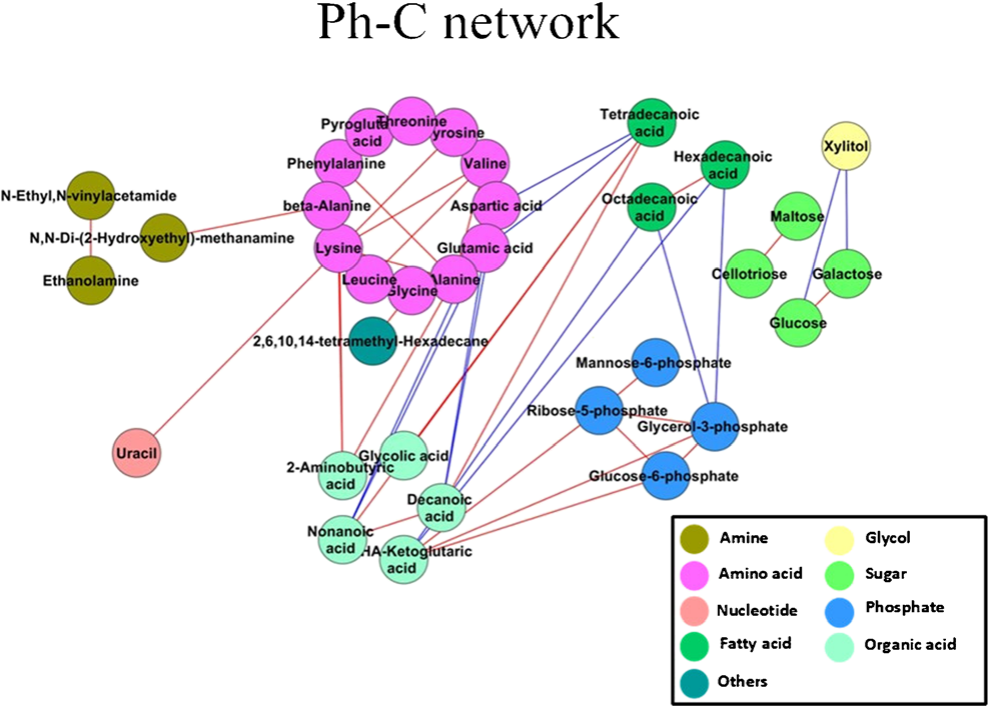
Correlation-based network analysis to identify *P*. *haitanensis*

Further screening of the correlations was performed, and 2 were determined to be significant with an *r* ^2^ ≥ 0.49 and a false discovery rate (FDR) ≤ 0.05 in the co-culture samples, among which 2 constituted positive correlations and 0 constituted negative correlations (Fig. 5). In the controls, there were only 41 significant correlations with an *r* ^2^ ≥ 0.49 and FDR ≤ 0.05 identified. Among them, 29 were positive correlations, while 12 were negative correlations. Organic acids and amino acids also played important roles in the metabolite correlations.

### KEGG pathway visualization

To visualize the metabolic pathways of the identified metabolites, a comprehensive metabolic map was constructed from the identified 72 metabolites with annotated pathway information in KEGG. These metabolites covered pathways or metabolisms that include aminoacyl-tRNA biosynthesis, citric acid cycle (TCA cycle), amino acid metabolism, sugar metabolism, and biosynthesis of secondary metabolites. We used “biosynthesis of plant secondary metabolites” as an example in Supplementary Fig. 6.

## Discussion

Seaweeds act as primary producers in marine ecosystems and engage in symbiosis with other organisms such as bacteria and fungi (Cooper and Smith 2015). The colonization of bacteria on the algal surface was displayed in the results, which indicate the physical basis for the interaction between algae and bacteria. Seaweed–bacteria interactions are mainly categorized into nutrient exchanges (Kouzuma and Watanabe 2015). A typical example of such an interaction is the supply of vitamin B_12_ by bacterial species to their seaweed partners in exchange for fixed carbon (Hodson et al. 2005; Helliwell et al. 2011). A metabolomics approach revealed the mutualistic relationship between the diatom *Thalassiosira pseudonana* and the bacterium *Dinoroseobacter shibae* when vitamin B_12_ was not limiting. *Dinoroseobacter shibae* also had a significant effect on the growth of *T. pseudonana* at the early stage of the co-culture, influencing the metabolites of *T. pseudonana* including several amino acids, fatty acids, and C4 sugars (Paul et al. 2012).

Nutrient exchange behaviors also occur in terrestrial plant–bacterium symbioses, such as the reliance of *Medicago truncatula* (alfalfa) on the nitrogen-fixing bacterium *Sinorhizobium meliloti*. In this symbiotic system, the plant excretes flavonoid molecules that stimulate the bacteria to produce oligopolysaccharides known as the Nod factor (Gage 2004), which in turn induces plant root cell division and curling of the root hairs to incorporate a symbiotic bacterial colony, and is thus important for the initiation of the symbiosis (Jones et al. 2007).

It has been hypothesized that metabolite-mediated interactions are essential for ecosystem functioning (Paul et al. 2012). In this study, the bacteria *Bacillus* sp. WPySW2 promoted the relative growth rate of *P*. *haitanensis* at 20 °C*.* Metabolites related to growth, energy storage, photosynthesis, and resistance-associated substances including organic acids, sugars, amines, glycols, and several amino acids of *P*. *haitanensis* were also altered when it was co-cultured with *Bacillus* sp. WPySW2, which could affect the physical status and environmental adaptation of the seaweeds in several ways (Yang et al. 2008).

### Sugars

The polysaccharides in *P*. *haitanensis* possess antioxidant and anti-aging activities (Zhang et al. 2003, 2010; Zhao et al. 2008) due to their free radical scavenging properties. Moreover, polysaccharides from *Pyropia yezoensis* have been found to have many physiological and nutraceutical activities, including antiviral, immunoregulatory, anticancer, anticoagulant, and antihyperlipidemic activities (Guo et al. 2007; Bhatia et al. 2013; Jiang 2014; Qian et al. 2014). Of the 72 identified metabolites, there were 4 polysaccharides (melibiose, cellotriose, maltose, and cellobiose) in the experimental group that exhibited changes in concentration (B-Ph; Fig. 3); however, only melibiose was found to be significantly upregulated (*p* < 0.05). Melibiose is an important disaccharide synthesized in the leaves during the photosynthesis of some plant species (Classen and Blaschek 1998) and is thought to be hydrolyzed from raffinose (Zhang et al. 2016).

The water-soluble polysaccharides produced by *Ulva* sp., such as rhamnose, xylose, glucose, mannose, and galactose, are used by epiphytic bacteria as a source of carbon and energy, resulting in the microbes gaining a competitive advantage in colonizing the host surface (Bruckner et al. 2011). The particulate acidic polysaccharides released by diatoms, such as transparent exopolymer particles (TEP), are abundant in the ocean and are often colonized by bacteria (Passow 2002). These acidic polysaccharides are used by active diatoms to recruit specific bacteria. The bacteria recognize the diatom and initiate attachment to the surface of the host, which provides the polysaccharides for the bacteria as a nutrient source.

Sugars and their derivatives have also been found to act as inducers for invertebrate settlement on the surface of seaweeds (Steinberg et al. 2001). These polysaccharides were also found in the metabolites of *P*. *haitanensis* and might provide the nutrients for the bacteria, which was substantiated by the fact that the bacteria could not survive in seawater but could grow well when they were co-cultured with the alga without additional nutrients (unpublished data).

### Glycols

Galactosylglycerol is a natural glycoside which is an important structural unit of glycoglycerolipid (Wei et al. 2013). The metabolite α-galactosylglycerol is a kind of isofloridoside and has an important role in regulating the osmotic balance to maintain physiological stasis in the golden brown alga *Ochromonas malhamensis* (Kauss 1973). Its structure is similar to isoflorid-oside/floridoside which has three isomeric forms, floridoside (α-d-galactopyranosyl-(-1 → 2)-d-glycer-ol), d-isofloridoside (α-d-galactopyranosyl-(-1 → 1)-d-glycerol), and l-isofloridoside (α-d-galacto-pyranosyl-(1 → 1)-l-glycerol) in the red alga *P*. *haitanensis* (Karsten et al. 1993). It is synthesized by β-galactosidase catalyzed reverse hydrolysis of galactose and glycerol (Wei et al. 2013).

On account of storage time being short in the cytosol, glycols are broken down into other carbohydrates such as red algal starch and the precursors of agar (Song 2014). In many single-cell red algae, galactosylglycerols are directly synthesized to the carbon precursors of cell wall polysaccharides (Li et al. 2002). In addition, this might be crucial for carbon transfer between some species of the cyanobacterial genus *Microcystis* and their hosts (Simon-Colin et al. 2004).

The red alga *P*. *haitanensis* in the intertidal zone is periodically exposed to air where it undergoes a variety of potentially stressful environmental changes, including abiotic stress (salinity, temperature, and seasonal factors) and biological stress by phycosphere microorganisms (Karsten and West 2000; Reed 1985; Reed et al. 1980). Compared with the control (Ph-C), the co-cultured *P*. *haitanensis* (B-Ph) contained significantly more galactosylglycerol in our study. It is suggested that the accumulation of galactosylglycerol in *P*. *haitanensis* could help maintain osmotic balance and facilitate adaptation to the environment when co-cultured with the phycosphere bacteria *Bacillus* sp.

### Phosphate

Glycerol-3-phosphate (G3P), an intermediate product of the fatty acid metabolism pathway, is involved in energy-producing reactions including glycolysis and glycerolipid biosynthesis and acts as a regulator of plant defense signaling. In this study, the concentration of G3P increased significantly in the co-cultured *P*. *haitanensis* (Fig. 3). G3P could be processed into floridosides, an important osmoprotectant in intertidal red algae, functioning in abiotic stress resistance reactions in *Pyropia* (Ye et al. 2013; Qian et al. 2015). However, glycoside metabolites were not detected by GC-MS in this study.

G3P, derived from glycerol in terrestrial plants, plays an important role in providing basal defense in the interaction of plant roots with fungal symbionts (Chanda et al. 2008). It was found that the glycerol content reduced while concomitantly increasing the G3P content in *Arabidopsis* infected by *Colletotrichum higginsianum* (Srivathsa et al. 2009). Strikingly, the accumulation of G3P in the plant tissues during interactions with microbes preceded the accumulation of other metabolites known to be essential for resistance responses (Chanda et al. 2011).

### Amino acids

Some amino acids are thought to be involved in stress resistance reactions in many algae. Proline is an essential constituent of most proteins and participates in many metabolic activities and is important in maintaining the osmotic balance of algal cells (Saha et al. 2014). Leucine, isoleucine, and valine are involved in deamination and transamination reactions connected with the tricarboxylic (TCA) cycle (Kakinuma et al. 2006). Ye et al. (2013) reported that the amounts of these amino acids increased in the high-temperature tolerant strains of *P*. *haitanensis*.

In this study, the concentrations of proline, tyrosine, threonine, leucine, isoleucine, valine, and phenylanin were strongly downregulated in *P*. *haitanensis* co-cultured with the bacteria (Fig. 3 and Table 1). Saha et al. (2012) reported that proline concentrations of 0.01 ng cm^−2^ or more were sufficient for a partial inhibition of all but the most resistant strains of *Cytophaga* sp. on the surface of *Fucus vesiculosus*.

The concentrations of alanine and serine were upregulated in the co-cultured *P*. *haitanensis* samples in this study. Alanine accumulation is a common response in plants and animals to a variety of stress conditions, such as anoxia and extreme temperatures (Eberlee and Storey 1988; Nissim et al. 1992). It has been proposed that alanine is a universal primary stress signal expressed by cells (Monselise et al. 2003). Compared with those at suitable temperatures, heat-stressed *P*. *haitanensis* were found to contain significantly more alanine, which is thought to act as an osmoprotectant in high-temperature tolerance reactions (Ye et al. 2013).

It has also been reported that alanine acts as a trigger to promote the probiotics of rice (*Oryza sativa*) to protect the plant against biofilms (Jones et al. 2011). However, serine is a precursor of G3P, which contributes to bacterial niche specificity through gene selection. This host metabolite has been found to further refine the response of bacteria to their environment and can dramatically affect the outcome of the host–pathogen interaction (Connolly et al. 2015). In addition, the human body contains serine at various extraintestinal sites, which ensures appropriate bacterial colonization (Connolly et al. 2016). Whether the accumulation of alanine and serine in *P*. *haitanensis* possesses a similar function of ensuring appropriate bacterial colonization requires further study.

The changes in the stress-related amino acids might indicate a reciprocal relationship between *P*. *haitanensis* and *Bacillus* sp. WPySW2 being constructed under such co-culture conditions. It was also previously demonstrated that free amino acids could enhance the primary production of seaweeds (Flynn and Wright 1986; Linares 2006), which might explain the promoted algal growth and bacterial colonization in co-culture.

### Fatty acids

Octadecanoic acid and hexadecanoic acid were significantly reduced in *P*. *haitanensis* when the phycosphere epibiotic bacteria were added (Fig. 3). Increasing evidence shows both C18 and C16 fatty acids and their derivatives act as signaling molecules, modulating normal and disease-related physiologies in some organisms (Kachroo and Kachroo 2009). Fatty acids (FAs) secreted by many algae act as antibacterial compounds to protect against unwanted and/or algicidal bacteria. The diatom *Navicula delognei* was found to produce hexadecatetraenoic acid and octadecatetraenoic acid, derived from octadecanoic and hexadecanoic acid, which displayed strong antibacterial activity against the pathogens *Staphylococcus aureus*, *Staphylococcus epidermidis*, *Proteus vulgaris*, and *Salmonella enterica* (Amin et al. 2012).

### Organic acids

Current knowledge shows that citric acid not only acts as an intermediate in carbon metabolism, but also as a key component in mechanisms that some plants use to cope with nutrient deficiencies, metal tolerance, and the operation of plant–microbe interactions (López-Bucio et al. 2000). Citric acid is an organic acid that has been proposed to be involved in those processes and is involved in energy production as photosynthetic intermediates in the TCA cycle (Jones 1998). There is some evidence that organic acid excretion could play an important role in phosphate solubilization and was first discovered when researchers found that the roots of certain plant species grown under phosphorus-deficient conditions contain higher concentrations of organic acids than non-stressed plants (Struthers and Sieling 1950; Bradley and Sieling 1953). It is well known that nitrogen and phosphorus are essential for plant growth, development and reproduction (López-Bucio et al. 2000). In this study, the concentration of citric acid decreased significantly in the co-cultured *P*. *haitanensis* samples (B-Ph; *p* < 0.05, Fig. 3), while the control (Ph-C) increased significantly. The result might indicate that the accumulation of citric acid enhanced resistance to stress, while co-culture could reduce environmental stress.

On one hand, the epiphytic bacteria *Bacillus* sp. WPySW2 changed the metabolite composition of seaweeds and promoted the growth of *P*. *haitanensis* in co-culture; on the other hand, it is envisaged that some metabolites produced by the phycosphere microbes act as a growth regulator. Some studies suggest that the genus *Bacillus* can promote plant growth directly through nitrogen fixation, phosphate solubility, and the production of phytohormones and 1-aminocyclopropane-1-carboxylic acid (ACC) deaminase and indirectly by the production of antagonistic compounds such as hydrolytic enzymes, siderophores, bacterial volatile compounds (BVCs), and a range of antibiotics (Parke and Doug 2001; Govindasamy et al. 2010; Zulma et al. 2012; da Costa et al. 2014; Chung et al. 2015; Bach et al. 2016). Thus, it is necessary to study microbial metabolism to comprehensively assess the symbiotic relationship between seaweeds and bacteria in particular.

In summary, the results demonstrated that *P*. *haitanensis* grew better when it was co-cultured with *Bacillus* sp. WPySW2 at 20 °C. Several main intracellular metabolites were downregulated or upregulated, which might facilitate bacterial colonization. Thus, a mutually beneficial relationship between *P*. *haitanensis* and epibiotic bacteria was identified as a contributor towards the maintenance of a stable phycosphere biological system. The combination of GC-MS-based metabolic profiling with multivariate analysis could potentially be a useful tool for discriminating the relationship between *P*. *haitanensis* and *Bacillus* sp. WPySW2 and determining the potential indicators of healthy algae–bacteria coexistence in ecosystems.

## Electronic supplementary material

ESM 1 (DOC 3802 kb)
