# Supplementary material for: Effect of the epiphytic bacterium Bacillus sp. WPySW2 on the metabolism of Pyropia haitanensis
Source: J Appl Phycol. 2017 Dec 2;30(2):1225–37. doi: 10.1007/s10811-017-1279-z (PMC5928181; doi:10.1007/s10811-017-1279-z)
Supplement: Supplementary file 1 — (DOC 3802 kb) [file 10811_2017_1279_MOESM1_ESM.doc]

**SUPPORTING INFORMATION**

The (TIC) of *Pyropia haitanensis*,72 identified metabolites, PCA and PLS-DA model, boxplot-visualizations and KEGG are included in the supplementary material.

**Figure Captions**

**Fig. S1** Representative GC-MS total ions chromatogram (TIC) of *Pyropia haitanensis*

**Fig. S2** The classification of the 72 identified metabolites in *P. haitanensis*

**Fig. S3** Principal component analysis (PCA) of metabolic profiles under control (Ph-C) and co-culture (B-Ph) conditions (eight biological replicates). (a) The score plot of PCA model; (b) The loading plot for the PCA model. t[1]/p[1]: for the first principal component; t[2]/p[2]: for the second principal component

**Fig. S4** PLS-DA of metabolic profiles under control (Ph-C) and co-culture (B-Ph) conditions (eight biological replicates). (a) The score plot of PLS-DA model; (b) The permutation test for the PLS-DA model

**Fig. S5** Boxplot-visualizations of significantly changed metabolites relative abundances in control (Ph-C) and co-culture (B-Ph) samples. (a) hexadecanonic acid; (b) octadecanoic acid; (c) leucine; (d) valine

**Fig. S6** Metabolic pathway map of “biosynthesis of plant secondary metabolites”. The map was constructed with metabolites annotated in KEGG. Metabolites were labeled with red

**Fig. S1**

**
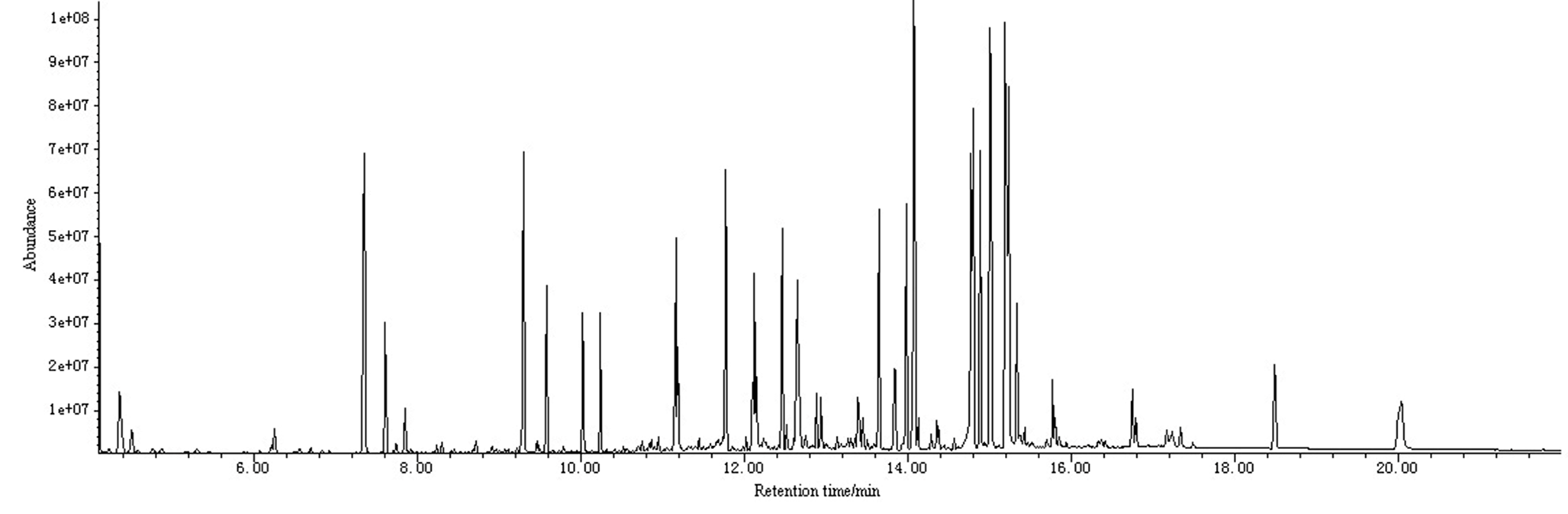
**

**Fig. S2**

**
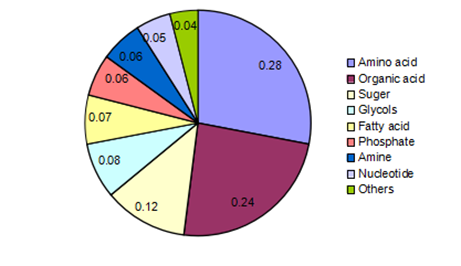
**

**Fig. S3**

**
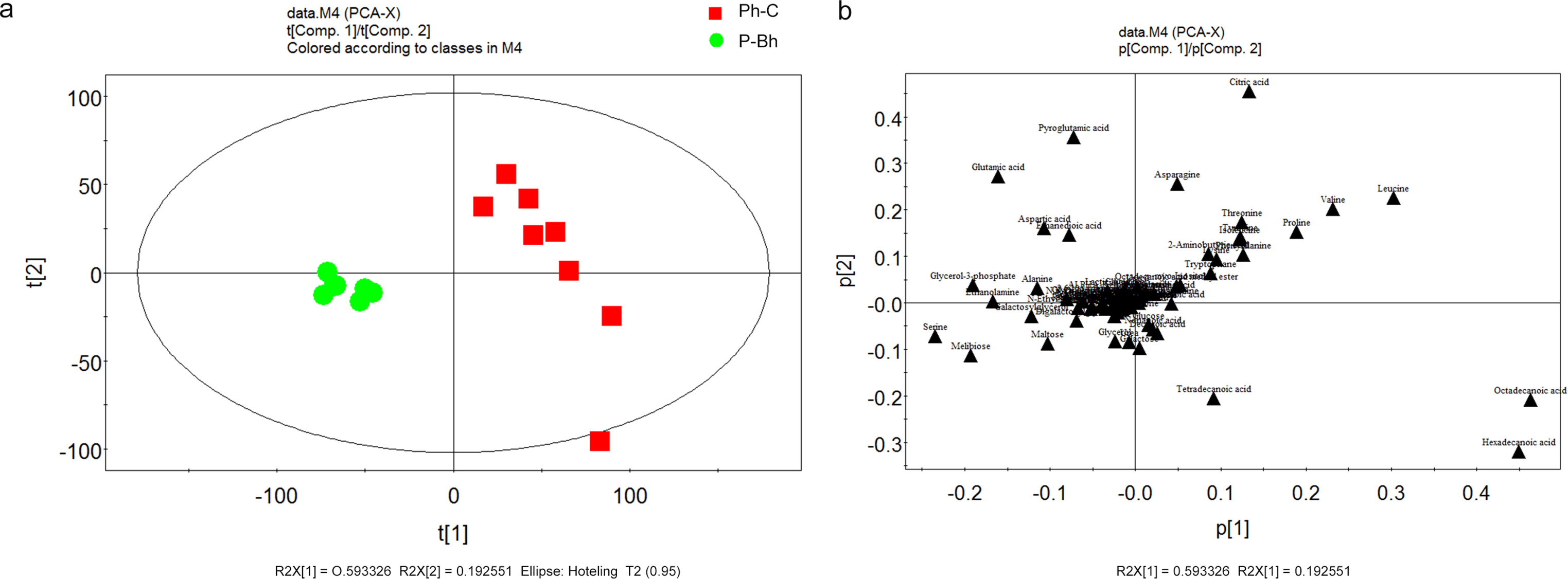
**

**Fig. S4**

**
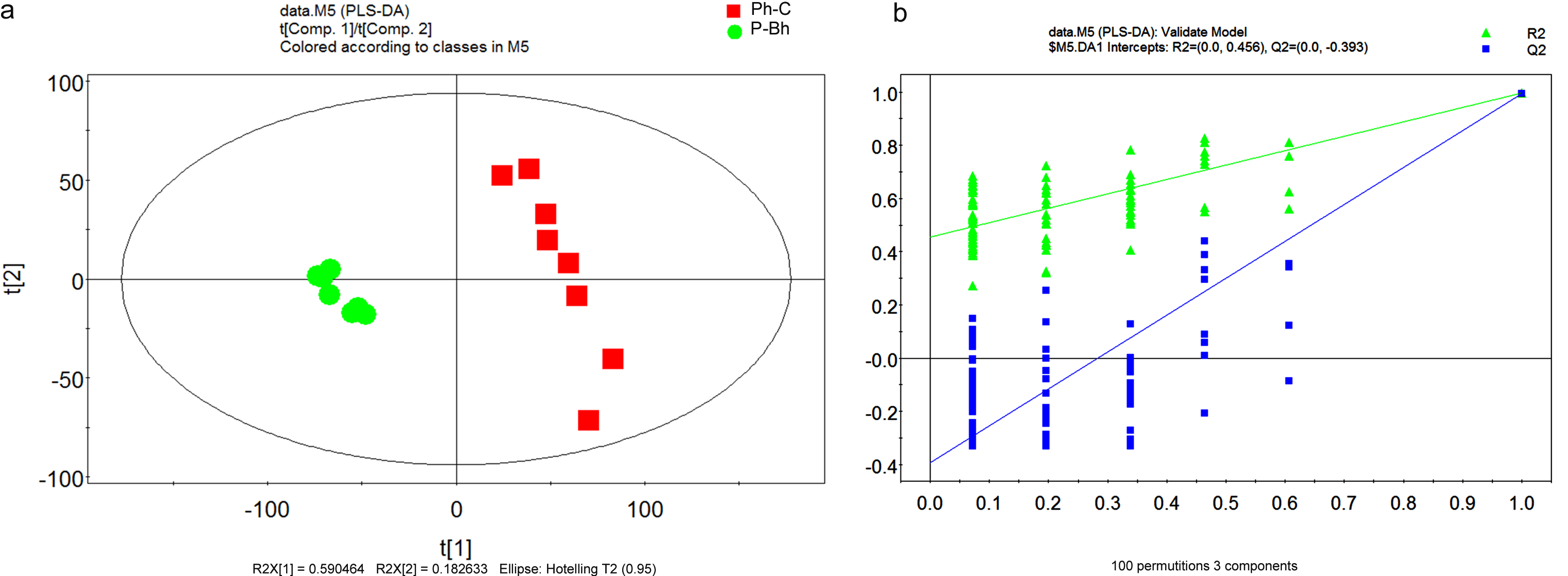
**

**Fig. S5
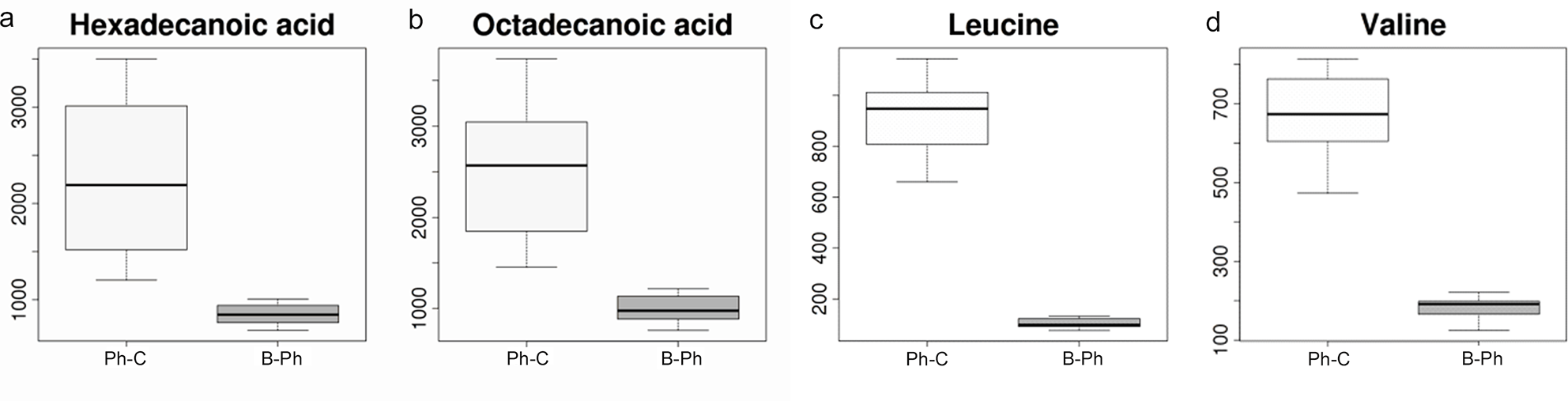
**

**Fig. S6**


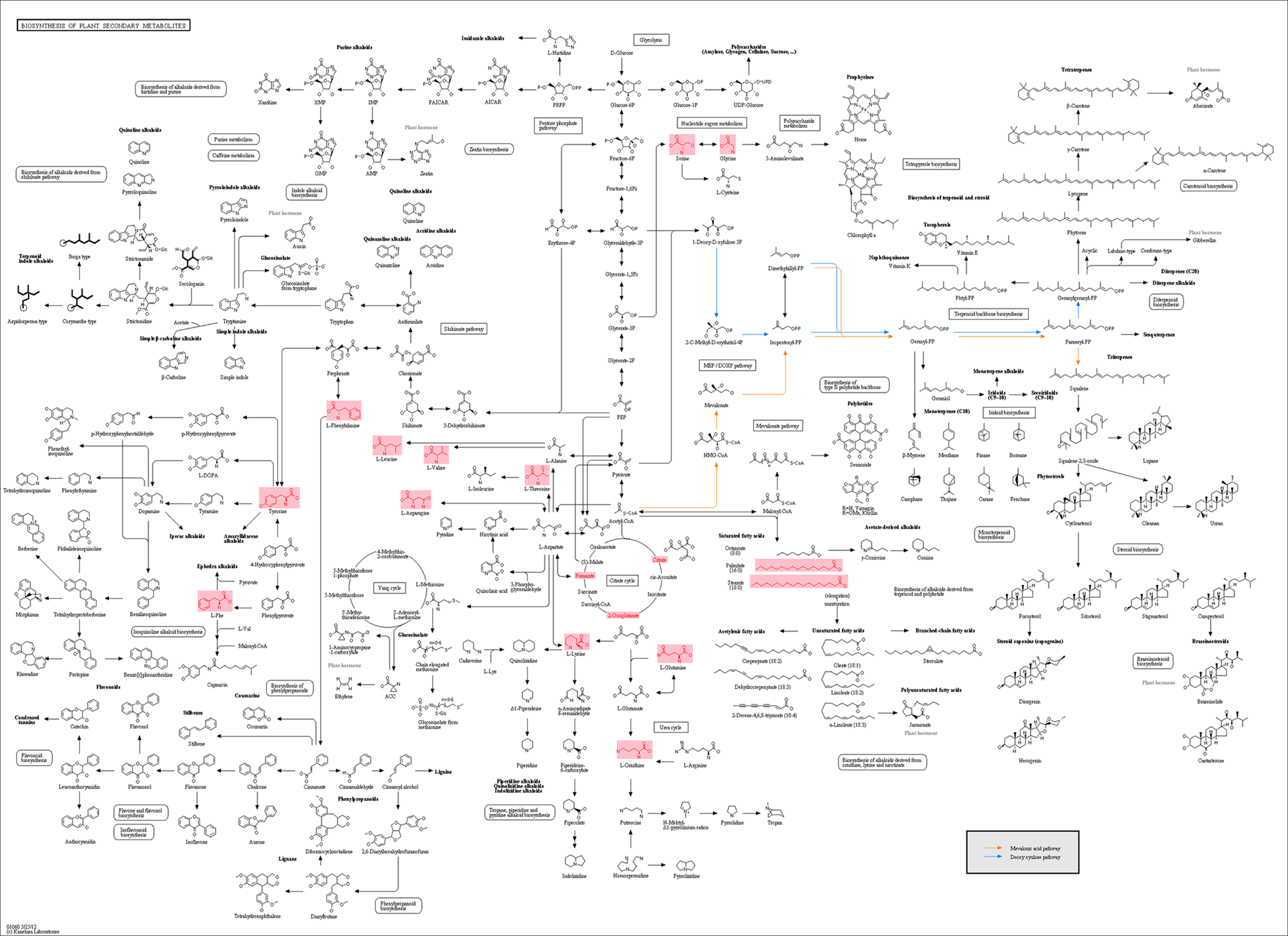


**Table 1** Annotated metabolites in pathway

| Pathway | description | compounds count | | Metabolites |
| --- | --- | --- | --- | --- |
| [map04976](../%5C%5C%5CD:%5Cwork%5Ccompany%5Cproject%5C谭紫菜%5CReport%5CKEGG%5Cpathway%5Cmap04976.html) | Bile secretion | 3 | Oxoglutaric acid(C00026) D-Glucose(C00031) CE(10:0)(C02530) | |
| [map00360](../%5C%5C%5CD:%5Cwork%5Ccompany%5Cproject%5C谭紫菜%5CReport%5CKEGG%5Cpathway%5Cmap00360.html) | Phenylalanine metabolism | 3 | L-Phenylalanine(C00079) L-Tyrosine(C00082) Fumaric acid(C00122) | |
| [map00290](../%5C%5C%5CD:%5Cwork%5Ccompany%5Cproject%5C谭紫菜%5CReport%5CKEGG%5Cpathway%5Cmap00290.html) | Valine, leucine and isoleucine biosynthesis | 3 | L-Leucine(C00123) L-Valine(C00183) L-Threonine(C00188) | |
| [map00480](../%5C%5C%5CD:%5Cwork%5Ccompany%5Cproject%5C谭紫菜%5CReport%5CKEGG%5Cpathway%5Cmap00480.html) | Glutathione metabolism | 3 | Glycine(C00037) Ornithine(C00077) Pyroglutamic acid(C01879) | |
